# A northern Chinese origin of Austronesian agriculture: new evidence on traditional Formosan cereals

**DOI:** 10.1186/s12284-018-0247-9

**Published:** 2018-10-11

**Authors:** Laurent Sagart, Tze-Fu Hsu, Yuan-Ching Tsai, Cheng-Chieh Wu, Lin-Tzu Huang, Yu-Chi Chen, Yi-Fang Chen, Yu-Chien Tseng, Hung-Ying Lin, Yue-ie Caroline Hsing

**Affiliations:** 1 Centre de Recherches Linguistiques sur l’Asie Orientale/Centre National de la Recherche Scientifique, INaLCO, 2 rue de Lille, 75007 Paris, France; 2 Institute of Plant and Microbial Biology, Academia Sinica, Taipei, 115 Taiwan; 3 Department of Agronomy, National Chiayi University, Chiayi, 600 Taiwan; 4 Institute of Plant Biology, National Taiwan University, Taipei, 106 Taiwan; 5 Taiwan International Cooperation and Development Fund, Taipei, 111 Taiwan; 6 Soil and Water Conservation Bureau, Council of Agriculture, Nantou, 540 Taiwan; 7 Department of Agronomy, Iowa State University, Ames, Iowa 50011-1085 USA; 8 Department of Agronomy, National Taiwan University, Taipei, 106 Taiwan

**Keywords:** Archaeology, Austronesian language, Domestication genes, Millet, Rice, Rice landraces, Taiwan Neolithic origins

## Abstract

**Background:**

Genetic data for traditional Taiwanese (Formosan) agriculture is essential for tracing the origins on the East Asian mainland of the Austronesian language family, whose homeland is generally placed in Taiwan. Three main models for the origins of the Taiwanese Neolithic have been proposed: origins in coastal north China (Shandong); in coastal central China (Yangtze Valley), and in coastal south China. A combination of linguistic and agricultural evidence helps resolve this controversial issue.

**Results:**

We report on botanically informed linguistic fieldwork of the agricultural vocabulary of Formosan aborigines, which converges with earlier findings in archaeology, genetics and historical linguistics to assign a lesser role for rice than was earlier thought, and a more important one for the millets. We next present the results of an investigation of domestication genes in a collection of traditional rice landraces maintained by the Formosan aborigines over a hundred years ago. The genes controlling awn length, shattering, caryopsis color, plant and panicle shapes contain the same mutated sequences as modern rice varieties everywhere else in the world, arguing against an independent domestication in south China or Taiwan. Early and traditional Formosan agriculture was based on foxtail millet, broomcorn millet and rice. We trace this suite of cereals to northeastern China in the period 6000–5000 BCE and argue, following earlier proposals, that the precursors of the Austronesians, expanded south along the coast from Shandong after c. 5000 BCE to reach northwest Taiwan in the second half of the 4th millennium BCE. This expansion introduced to Taiwan a mixed farming, fishing and intertidal foraging subsistence strategy; domesticated foxtail millet, broomcorn millet and *japonica* rice; a belief in the sacredness of foxtail millet; ritual ablation of the upper incisors in adolescents of both sexes; domesticated dogs; and a technological package including inter alia houses, nautical technology, and loom weaving.

**Conclusion:**

We suggest that the pre-Austronesians expanded south along the coast from that region after c. 5000 BCE to reach northwest Taiwan in the second half of the 4th millennium BCE.

**Electronic supplementary material:**

The online version of this article (10.1186/s12284-018-0247-9) contains supplementary material, which is available to authorized users.

## Background

In this paper we investigate the contribution of early Austronesian agriculture, especially rice cultivation, to the question of Austronesian origins. Austronesian is a largely insular language family that extends from southeast Asia to the eastern Pacific and Madagascar (Additional file 1: Figure S1). The main traditional cereals cultivated by the modern Austronesians in Taiwan, an island thought to be the Austronesian language family homeland, are upland rice, foxtail millet and broomcorn millet. Rice (*Oryza sativa japonica*) is believed to have been domesticated in the Yangtze basin c. 6000 BCE (Deng et al. 2015) and the millets in north China, also c. 6000 BCE (Bettinger et al. 2010). In Taiwan, rice and foxtail are ubiquitous. Culturally, foxtail *(Setaria italica)* has sacred status among most tribes (Fogg 1983). Broomcorn millet (*Panicum miliaceum*) is limited to mountain areas in the north of the island, having been abandoned by many groups in favor of introduced cereals such as sorghum and maize. From the seventeenth century to modern times, one finds specific references to rice and foxtail millet grown by aboriginal populations in documents produced by western visitors to Taiwan (Happart 1650; Esquivel 1633) and in eighteenth-century Chinese-Siraya bilingual land contracts (Li and Durbin 2010). Before the seventeenth century the millets are archaeologically present almost continuously from 1400 CE to 2800 BCE (Table 1). Due to their tiny size as compared to rice, millet grains can barely be detected unless flotation techniques are used; when detected, they are difficult to determine without microscopy. At several sites, the millets have not been determined to genus and species levels. A decrease in the amount of millet grains in the terminal Tahu culture (c. 1600–800 BP) is compatible with accidental variations in the amount of available evidence. The earliest and most compelling evidence for co-cultivation of the three cereals is from Nan Kuan Li East (NKLE), a neolithic site on the southwest coast of Taiwan dated to 3000–2300 BCE: there, grains of all three cereals occur together in large quantities (Tsang et al. 2017). The low frequencies of dental infection reported in NKLE skeletons indicates a diet low in starches and sugars (Pietrusewsky et al. 2013). That is, farming only represented one aspect of early Formosan subsistence strategy, as hunting, fishing and coastal foraging are also well evident (Li 2013).

**Table 1:** Long-term persistence of rice-and-millet agriculture in Taiwan

Culture		Tapenkeng	Niuchoutzu	Tahu		Fantsaiyuan	Niaosong
				late	terminal	terminal	early
Excavated site		NKLE	YHF	SL	SL	HLL	SL
Dates (BP)		5000–4200	3800–3300	2350–1800	1800–1400	1600–800	1400–900
	Foxtail millet	+++++	++++	++	+	+	+++
	Broomcorn millet	+++++					
	Rice	++++	+++++	+++	+++	+++++	+++

NKLE: Nan Kuan Li East, southern Taiwan (Tsang, 2012); YHF: You Hsien Fang, southern Taiwan (Tsang, 2012); SL: Siliao, southern Taiwan (Liu et al. 2011); HLL: Hui Lai Li, central Taiwan (Huang et al. 2006). Grain count: + 1–10; ++ 10–100; +++ 100–1.000; ++++ 1.000–10.000; +++++ 10.000–100.000

There are strong linguistic reasons for why cultivation of rice and foxtail and broomcorn millet in Taiwan cannot have been interrupted from at least 2000 BCE. It is generally agreed that the Austronesian languages outside of Taiwan (‘Malayo-Polynesian’) were founded in a single out-of-Taiwan migration event, c. 2000 BCE. Phonetically matching words for each of the cereals occur in both the Austronesian languages of Taiwan and outside of Taiwan. The regular pattern of correspondence in their vowels and consonants indicates that the Taiwanese and non-Taiwanese words are vertically inherited from a single prototype, which cannot be more recent than the out-of-Taiwan event. The ancestral Proto-Austronesian words for foxtail, broomcorn and rice have been reconstructed as *beCeŋ, *baCaR and *pajay, respectively (Wolff 2010; Tsuchida 1976; Blust and Trussel 2016; Shomura et al. 2008). In addition, the ancestral Proto-Austronesian language has also been shown to have had words for the boat, house, hunting, fishnet, domesticated dog, and field (Wolff 2010; Tsuchida 1976; Blust and Trussel 2016).

### Three models of Taiwan Neolithic origins

**Fig. 1:**
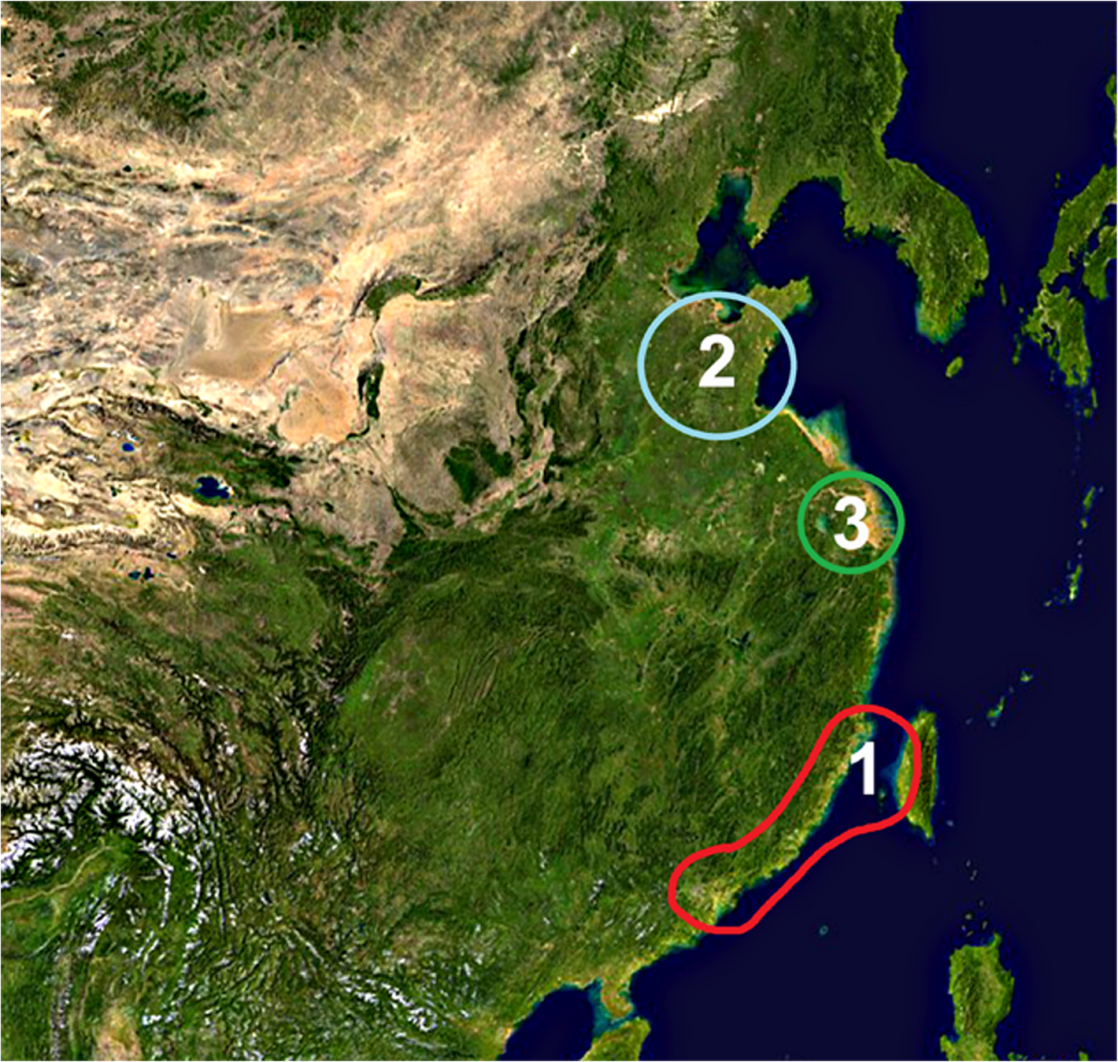
Mainland origins of the Taiwanese Neolithic according to three models. Blue: Northeastern Seaboard (NES, 2); green: Lower Yangtze (LY, 3); red: Chinese interaction sphere (CIS, 1). The northeastern Asia image was downloaded from https://commons.wikimedia.org/wiki/File:Asia_satellite_orthographic.jpg originally from NASA

In the past, archaeologists have proposed three main models of the origins of the Taiwanese Neolithic (Fig. 1). (1) One general model of Chinese neolithization (‘Chinese Interaction Sphere’, CIS) proposes for Taiwan an indigenous neolithic transition in coastal south China, becoming part of a network of cultural interactions across Neolithic groups in China in 4000–3000 BCE (marked as red in Fig. 1) (Chang, 1986). Evidence includes the existence of a pre-agricultural stage in Tapenkeng culture, the oldest ceramic culture in Taiwan (Hung and Carson Mike 2014), and a similarity between the earliest ceramic shapes in Tapenkeng culture and in the Pearl River Delta at similar or older dates (Tsang 2005a, 2005b). One variant of this model (CIS-1) assumes an independent domestication of rice in Taiwan (Li 1976; Li 1981). Another variant (CIS-2) sees agriculture being adopted as a whole (rice and millets) c. 2800 BCE through cultural interaction with Neolithic groups further north or inland (Hung and Carson Mike 2014; Deng et al. 2017). (2) An entirely different model (Northeastern Seaboard; NES) argues from shared cultural and material traits for the southward expansion of a neolithic population from the northeastern China coast, especially the Shandong peninsula (marked as blue in Fig. 1) (Ling 1951; Chang 1959). This model in its original form was abandoned after K. C. Chang, its original proponent, elaborated the CIS model; it has been revived under the linguistic proposal assigning a common origin to the Austronesian and Sino-Tibetan families (Sagart 2005; Sagart 2008): according to this model, a southward expansion along the China coast brought the pre-Austronesian farmers out of Shandong and into Taiwan between 5000 and 3500 BCE. (3) A third model (Lower Yangtze; LY) was formulated within the “Farming/language theory” when lower Yangtze neolithic sites such as Hemudu were thought to hold the earliest domesticated rice in the world and before millet was archaeologically discovered in Taiwan. That model essentially aims at explaining the appearance in Taiwan of the Proto-Austronesian as a result of a demographic expansion fueled by the domestication of rice in the lower Yangtze and Hangzhou Bay area (marked as green in Fig. 1) (Bellwood 1997; Blust 1996).

We examine the compatibility and viability of these models using linguistic and genetic data of crop species. Two research questions have a direct bearing on the issue:

1. Which cereal, if any, was culturally the more central of foxtail millet, broomcorn millet and rice in pre-modern Taiwan? Rice would favor the LY model, since Lower Yangtze sites show a largely rice-based subsistence strategy. The NES model is more compatible with a less central role of rice, as the same three cereals as in Taiwan were cultivated in the Shandong area before the onset of the Formosan Neolithic, and rice there is the least prominent of the three.
2. Was Taiwanese rice independently domesticated? A positive answer would support the CIS-1 model: under both the NES and LY models, there was a single rice domestication event in East Asia. On the contrary, if the traditional rice landraces of the Formosan Austronesians shared the same domestication traits with other East Asian rice, that would exclude an independent Neolithic transition in south China/Taiwan.

We address the first question through linguistics. As many as eight unanalyzable words referring exclusively to rice are claimed to have existed in the ancestral Austronesian language (Blust and Trussel 2016), in contrast to the supposedly more restricted vocabulary of millet. This has led to notions that rice was more central to the Austronesian food production strategy than the millets. Fieldwork carried out by us among the Formosan Austronesians—whose languages represent the highest-order branches of the family—allows us to reevaluate that claim.

We address the second question through genetic characterization of a set of sixty traditional upland rice accessions collected by Japanese investigators around 1900 and successfully cultivated by us at the Academia Sinica campus in Taiwan. We aimed to assess whether these landraces can be taken as descendants of the earliest Austronesian rice; establish by DNA sequencing whether they have undergone the domestication-related mutations present in all other Asian rice; and establish their phylogenetic position among Asian rices.

## Results

### The early Austronesian vocabulary of domesticated cereals

In the summer and fall of 2017, we collected agricultural vocabulary from the main Austronesian-speaking tribes in Taiwan. The main target was foxtail millet. In a significant number of cases, informants' responses to our questions on the native words for ‘cooked foxtail’, ‘dehusked foxtail grains’, ‘chaff of foxtail’, ‘mortar used for foxtail millet’, ‘germinated grain of foxtail’, ‘foxtail seed for planting’, ‘flour of foxtail’, and ‘to pound foxtail grains’ (Table 2) were the same words as those presented as referring only to rice since the earliest Austronesian times in a major repository of Austronesian vocabulary (Blust and Trussel 2016). Had the data we collected been taken into account, these words would have been reconstructed with generic meanings: ‘cooked grain’, ‘dehusked grains’, ‘chaff’, ‘mortar’, ‘germinated grains’, ‘seed for planting’, ‘flour’, and ‘to pound’. Evidently, the rice-specific meanings were obtained by earlier investigators as responses to rice-specific question such as ‘what is the term for ‘cooked rice’? ’, without the corresponding questions about millet being asked. We conclude that the apparent prominence of rice-specific vocabulary in Proto-Austronesian is the result of an ascertainment bias: there are no linguistic grounds to conclude to a predominance of rice over foxtail millet in Taiwan Neolithic. Linguists have been the victims of an “obsession with rice”, just like southeast Asian archaeologists (Castillo 2017).

**Table 2:** Formosan language evidence for the meaning of eight reconstructed agricultural words

Rice-specific PAN meaning acc. to (Blust and Trussel 2016)	PAN form	Evidence cited in (Blust and Trussel 2016) in support of rice-specific meaning	Foxtail millet-related cognate forms from our fieldwork
cooked rice	*Semay	Amis *hmay* ‘cooked rice’ Kavalan *mːay* ‘cooked rice’ Pazeh *sumay* ‘cooked rice’	Amis (Dulan) *hmay* ‘cooked grain, as of foxtail’ Kavalan *mmay* ‘cooked grain, as of foxtail’ Kaxabu *su'maj*‘cooked grain, as of foxtail’
rice between harvesting and cooking; husked rice	*beRas	Amis *felac* ‘milled rice grain’ Atayal *buax ‘*hulled, uncooked cereal; grains (of rice or millet)’ Kanakanabu *və’əra* ‘husked rice’ Kavalan *beRas* ‘rice that has been harvested but not yet cooked’ Paiwan *vat* ‘seed, kernel, grain; testicles’ Puyuma (Tamalakaw) *veRas* ‘husked rice’ Rukai (Maga) *bósə* ‘husked rice’ Rukai (Tona) *bəʔásə* ‘husked rice’ Saaroa *ə-vəraə* ‘husked rice’ Tsou *fərsə* ‘husked rice’	Amis (Dulan) *flac ‘*dehusked grain of foxtail’ Atayal *vuax* ‘dehusked grain, as of foxtail’ Kanakanabu *vura ‘*dehusked grain, as with foxtail’ Kavalan *vχas* ‘dehusked grain, as of foxtail’ Paiwan (Daniao, Taiban, Dewen) *vat* ‘dehusked grain, as of foxtail’ Rukai (Dewen) *‘bəratə* ‘dehusked grain, as of foxtail’ Sediq *beras* ‘dehusked grain, as with foxtail’
rice bran/husk	*qeCah	Amis *ʔtah* ‘chaff’ Proto-Rukai *eca ‘husk of rice’ Puyuma (Tamalakaw) *HeTa* ‘rice husk and bran’	Amis tah ‘chaff, as of foxtail millet’ Rukai (Taromak) eca ‘chaff, as of foxtail millet’
rice mortar	*iŋsuŋ	Kavalan *iŋsuŋ* ‘mortar’	Kavalan *insuŋ* ‘dehusking/crushing mortar, used for foxtail millet’
rice seedling	*bunabun	Paiwan *vunavun* ‘seedlings of grains or grasses’	Rukai (Dewen) *bunabunu* ‘germinated grain, seedling, as with foxtail millet’
seed rice, rice set aside for the next planting	*bineSiq	Bunun *binsiq* ‘seed preserved (as millet seed)’ Puyuma *bini* ‘seed of grains’ Saisiyat *binSi* seed of grains Thao *finshiq* to sow, scatter seed in planting	Saisiyat *binʃiʔ* seed for sowing (as of foxtail millet)’ Thao *kamar a vavinçeq* ‘foxtail millet seeds for sowing’ Bunun *binisjɪq* ‘seed for sowing (as of foxtail millet)’
sticky rice cake	*qemu	Amis *ʔəmu* ‘New Year's cake, made of sweet sticky rice’ Paiwan *qemu* ‘something that has been beaten in a mortar, as millet into flour’ Puyuma *Hemu* ‘flour’ Thao *qmu* ‘sticky rice; sticky rice cake’	Paiwan (Daniao, Taiban) *q < in > əmu* ‘flour, as of foxtail millet’
to pound rice	*bayu	Bunun *ma-bazu* ‘to pound’	Bunun *maɓaðu* ‘to pound grain to remove seed coat, as with foxtail millet’

### Early Formosan rice is phenotypically highly diverse

Additional file 2 Figure S2 illustrates the morphology of seed and caryopsis for all 60 accessions plus two modern varieties for comparison. Thirty-five accessions are awnless; eleven have awns 4 cm or longer. The rest have short awns, under 3.5 cm in length. Most accessions have white caryopsis, four have red caryopsis. Thus, judging solely from seed morphology, our collection includes a very large amount of phenotypic variation. In our previous work, we showed that there are also very large differences in flowering response to photoperiod (Wei et al. 2016a, 2016b). This makes our collection well-suited to the study of early domestication-related genes and of phylogenetic relationships with other rice accessions, including modern varieties, other landraces and wild rice.

### Three kinds of Formosan landraces

**Fig. 2:**
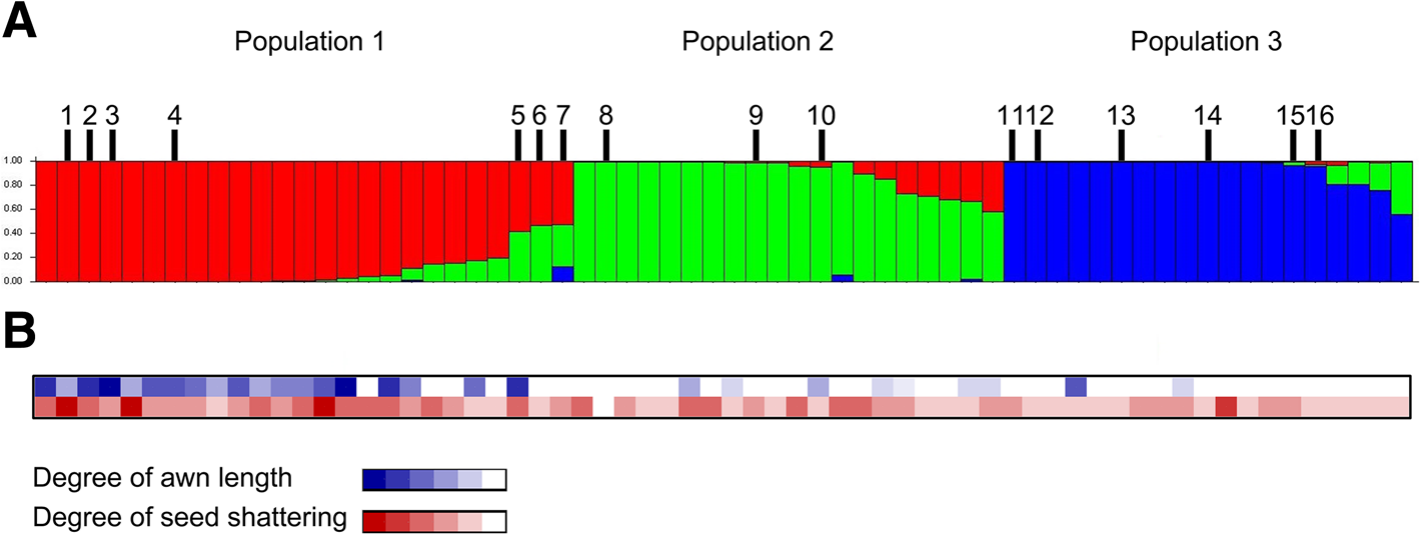
Classification of 60 Formosan upland rice accessions and 4 control varieties using STRUCTURE v2.3.1 with K set at 3. Panel **a**. Population 1 (red) primitive *japonica*; population 2 (green): relatively modern *japonica*; population3 (blue): *indica*. Numbers above the main graph identify accessions discussed in the text: 1, Nakairitsu; 2, Kabotsumame; 3, Matara; 4, Chuan No4; 5, Bohai; 6, Purahaitairin; 7, Montana; 8, Nipponbare; 9, Nakabo; 10, Muteka; 11, Ragarasu; 12, Tangengenrankatsu; 13, Tapopuri; 14, IR64; 15, Nobohai; 16, Parahainakoru. Panel **b**. Degree of awn length (blue; white indicates no awn) and seed shattering (red; white indicates no shattering) of each accession. The list of accessions is shown in Additional file 1: Table S4, and the seeds are available at National Germplasm Center, Taiwan Agriculture Research Institute, Taiwan and T.T. Chang Germplasm Center, International Rice Research Institute, the Philippines

Several methods are available to distinguish *japonica* and *indica* rices. We relied on two molecular markers, ORF100 (Kanno et al. 1993) and RBIP (Vitte et al. 2004) to check the type of each accession. Among 60 accessions investigated, about 45 were *japonica* and the rest *indica*. The population structure within our collection was inferred using STRUCTURE v2.3.1 (Evanno et al. 2005). The classification of accessions into populations by the model-based method is shown in Fig. 2a with K value set at 3. Two modern varieties, Nipponbare for *japonica* and IR64 for *indica*, as well as two landraces grown in Taiwan since the seventeenth century were used as internal standards. Populations 1, 2, and 3 contained 25, 19, and 16 Formosan landraces, respectively, in addition to two modern varieties and two other landraces in the analysis. The degree of awn length and shattering of each accession is illustrated in Fig. 2b. Many accessions among the red-color population (Population 1) are long-awned and shattering. All are *japonica*. Hence, this population may be characterized as primitive *japonica*. The modern variety Nipponbare was grouped with Population 2 (green color). Two Formosan rice accessions, Nakabo and Muteka, previously shown to be introgression donors to the modern megavariety Taichung 65, were also classified as Population 2: both are temperate *japonica* (Wei et al. 2016b). A few accessions within Population 2 have short or no awns and about half of them are low-shattering. Thus, this group can be characterized as less primitive *japonica*. The modern variety IR64 and the two Formosan landraces Pai-K'o-Tsao-Tzu and O-Loan-Chu were classified as Population 3 (blue color). None of this group's members have long awns and most are low-shattering. All the accessions in Population 3 are *indica*. Thus, the blue-color population contains less primitive *indica* rice. That the most primitive of our Formosan rice accessions belong to *japonica*, while all our Formosan *indica* accessions are quite modern implies that the earliest Formosan rices were of the *japonica* type.

### The more primitive Formosan landraces belong to *japonica*

**Fig. 3:**
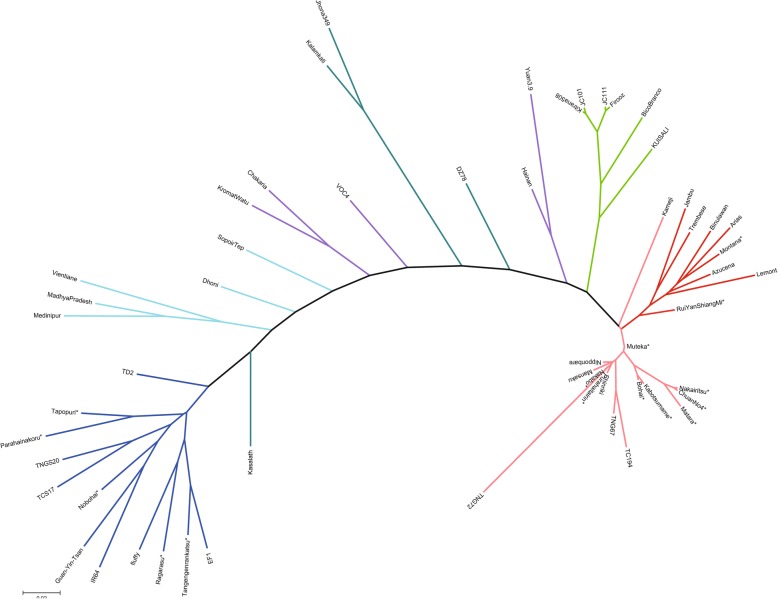
Phylogeny of the 55 rice accessions. Red: *japonica* (dark red: tropical *japonica*); green: *aromatic*; cadetblue: *aus*; cyan: wild rice (*Oryza nivara*); blue: *indica*; purple: wild rice (*Oryza rufipogon*). Neighbor-joining phylogenetic tree based on all SNPs of the 55 accessions in Additional file 1: Table S4. Bootstrap values determined with 1000 samples are shown. The scale bar indicates the simple matching distance. Aboriginal Formosan accession names are followed by an asterisk

To reveal the genetic relationships of Formosan rices with other rice groups, we performed a phylogenetic analysis (see the section on materials and methods below). To that end, we selected fourteen accessions with different awn lengths and shattering degree from the three populations in our STRUCTURE analysis. To these, we added one primitive Formosan landrace collected from an aboriginal village in 2014: Rui Yan Shiang Mi. This landrace has purple palea and lemma, red caryopsis, long awns and is shattering. The names of these accessions and their early domestication-related phenotypes are shown in Additional file 3: Table S1. We included in the phylogenetic analysis published NGS data for forty more accessions: five *O. nivara*, five *O. rufipogon*, seven temperate *japonica*, six tropical *japonica*, seven *indica*, four *Aus*, and six *aromatic* rice accessions for comparison. The resulting phylogeny is shown in Fig. 3.

All *japonica* accessions fall within a single cluster, colored in two different shades of red in Fig. 3. Accessions colored in lighter red include modern temperate *japonica* rices such as Nipponbare from Japan and TC194, TNG67 and TNG72 from Taiwan; traditional temperate *japonicas* from Japan such as Kameji, Mansaka and Shinriki; and traditional Formosan accessions such as Nakabo, Purahaitairin, Muteka, Nakairitsu, Chuan No4, Matara, Kabotsumame, Bohai, Montana and Rui Yan Shiang Mi. The traditional *japonica* landraces from the Philippines and Indonesia, generally classified as tropical *japonica*, occur in a single subcluster, colored in dark red. The awnless Formosan landrace Montana also occurs in that subcluster. It is still unclear whether the Formosan *japonica* landraces should be classified as temperate, tropical, or intermediate, but it is relevant to note that six Formosan *japonica* landraces forming a subcluster in Fig. 3: Muteka, Nakairitsu, Chuan No4, Matara, Kabotsumame, and Bohai, have markedly primitive characteristics: in particular relatively long awns (2–6 cm) and a relatively high degree of shattering. Moreover, the nested position of the tropical subcluster within the broader *japonica* cluster suggests that the tropical *japonicas* of the Philippines and Indonesia arose as an adaptation of temperate or Formosan *japonicas* to tropical conditions, rather than the reverse. Specifically, Fig. 3 suggests that certain Formosan *japonica* landraces like Rui Yan Shiang Mi are intermediate between Formosan *japonicas* and the tropical *japonicas* of the Philippines and Indonesia. This makes good linguistic sense since all the languages of the Philippines and Indonesia belong to the Malayo-Polynesian branch of the Austronesian language family, and Malayo-Polynesians are believed on linguistic and archaeological grounds to have expanded south of Taiwan in a single sea-borne migration c. 4000 BP. Under the phylogeny in Fig. 3, the first Malayo-Polynesian-speaking rice farmers travelled south with the *japonica* varieties cultivated in southern Taiwan c. 4000 BP: these included one landrace ancestral to Rui Yan Shiang Mi and to the tropical *japonicas* of the Philippines and Indonesia. That variety proved especially successful in the new environment, giving rise to the modern tropical *japonica* rices of the Philippines and Indonesia. We expect that if more *japonica* landraces from other Austronesian-speaking areas, such as Madagascar, were subjected to phylogenetic analysis, they would fall into the same tropical *japonica* subcluster.

All *indica* accessions occur within a single cluster, colored in dark blue in Fig. 3. This includes modern *indica* varieties: IR64, TCS17, TNGS20, and local landraces such as Fluffy and EF1. Five Formosan accessions: Tangengenrankatsu, Nobohai, Parahainakoru, Ragarasu, Tapopuri, fall within the same cluster. All five are awnless and have a low degree of shattering. We regard them as *indica* rices introduced to Taiwan in historical times, much later than the *japonica* landraces.

### The haplotypes of early domestication genes in Formosan rice

We used NGS data to study the genes controlling awn, shattering, caryopsis color, and plant type in Formosan landraces, including *An1* (Luo et al. 2013), *An2* (also known as *LABA1*) (Gu et al. 2015; Hua et al., 2015), *Sh1* (Konishi et al. 2006), *Sh4* (Huang et al. 2006), *Rc* (Sweeney et al. 2006), *PROG1* (Jin et al. 2008) and *Lg1* (Ishii et al. 2013; Zhu et al. 2013). It has been suggested that these are early domestication genes (Meyer and Purugganan 2013; Olsen and Wendel 2013). A recent study (Choi and Purugganan 2018) confirms that *An2* (*LABA1*), *PROG1* and *Sh4* are early domestication genes. Choi and Purugganan argue that de novo domestication occurred only once, in *japonica*, with subsequent transfer of the domestication alleles to *indica* rice through introgression. In the supplementary materials, where neighbor-joining trees for low-diversity genomic regions are shown, Choi and Purugganan claim that several other genes, such as *Lg1*, are also early domestication genes (Choi and Purugganan 2018).

Loss-of-function *an1* and *an2* cause shortened awns (Luo et al. 2013, Gu et al. 2015; Hua et al., 2015); seeds with loss-of-function *sh1* and *sh4* are low- or non-shattering (Konishi et al. 2006; Huang et al. 2006), seeds with loss-of-function *rc* have white instead of red caryopses (Sweeney et al. 2006), loss-of-function *lg1* causes closed instead of spread-out panicles (Ishii et al. 2013, Zhu et al. 2013), and plants with loss-of-function *prog1* have straight instead of spread-out stature (Jin et al. 2008). The relevant gene loci, changes in sequences and phenotypes are listed in Additional file 3: Table S2.

Table 3 summarizes the sequence changes in early domestication genes among Formosan rice accessions. All 15 Formosan rice accessions have the same sequence changes as Nipponbare for *prog1* and *Oslg1*: the mutation from A to T in the *prog1* gene and from G to A in the *lg1* gene both lead to loss-of-function of these two genes. These haplotypes coincide well with their plant stature (from wide-open to relatively closed) and panicle phenotype (from open to closed). As for the shattering-related genes, all the functional SNPs in the loss-of-function *sh4* allele (i.e., mutation from G to T) occurred in all cultivar accessions tested, leading to a less shattering phenotype than in the wild rice species. However, the functional SNP of loss-of-function *sh1*, a mutation from G to T, occurred in Nipponbare only. This allele is not present even in TNG67 and IR64, the modern *japonica* and *indica* accessions used as controls in the study. It was demonstrated earlier that this mutation did not occur in the early stages of domestication (Kovach et al. 2007). In fact this loss-of-function allele is limited to some accessions in Japan and Korea. For *an1*, the gene controlling the presence and length of an awn, all 10 Formosan *japonica* accessions contain the same sequences as Nipponbare. That is, a TE was inserted into the gene causing its loss-of-function. Similar sequence changes are also present in another modern variety Tainung 67. Among our five Formosan *indica* accessions, however, four out of five have another haplotype —a 1-bp deletion— which also led to a loss-of function phenotype. Parahainakoru, the remaining *indica* accession, on the other hand, has both the TE insertion and the 1-bp deletion. For *an2*, another awn-controlling gene, 9 out of 10 Formosan *japonica* accessions contain a 29-bp insertion, similar to the two modern varieties Nipponbare and Tainung 67. Montana, an awnless Formosan accession clustering with tropical *japonica* rice in our phylogeny (Fig. 3), has both the 29-bp insertion and a 1-bp deletion. This 1-bp deletion in Montana may be introgressed from *indica*, since all *indica* accessions tested contain both the 29-bp insertion and 1-bp deletion. Either of the 29-bp insertion or the 1-bp deletion cause loss-of-function in the *an2* gene, leading to a shorter awn, or no awn at all. The awns in these accessions are much shorter than in most wild rice (about 15–30 cm). Only three accessions have red caryopsis; the rest have white caryopsis. Like wild rice, Rui Yan Shiang Mi and Kasalath do not contain the 14-bp deletion in *Rc*, and all three have a red caryopsis, indicating a functional *Rc*. All other accessions have the 14-bp deletion leading to the loss-of function allele and white caryopsis.

**Table 3:** Summary of sequence changes in early domestication-related genes

Sample	Sub-spp	Awn length^a^	Shattering degree^b^	*Prog1*	*OsLG1*	*Sh4*	*An1*	*An2* (*LABA1*)	*RC*	*Sh1*
				SNP	SNP	SNP	TE ins or 1-bp del	1-bp del or 29-bp ins	14-bp del	SNP
Bohai	J	+++	+++	T	A	T	TE ins	29-bp ins	14-bp del	G
Chuan No4	J	+++	++	T	A	T	TE ins	29-bp ins	14-bp del	G
Kabotsumame	J	+++	+++	T	A	T	TE ins	29-bp ins	14-bp del	G
Matara	J	++	+++	T	A	T	TE ins	29-bp ins	14-bp del	G
Montana	J	–	++	T	A	T	TE ins	Both	14-bp del	G
Muteka	J	++	++	T	A	T	TE ins	29-bp ins	No deletion	G
Nakabo	J	–	++	T	A	T	TE ins	29-bp ins	14-bp del	G
Nakairitsu	J	++	++	T	A	T	TE ins	29-bp ins	14-bp del	G
Purahaitairin	J	+	+	T	A	T	TE ins	29-bp ins	14-bp del	G
RuiYan	J	++	++	T	A	T	TE ins	29-bp ins	No deletion	G
Nipponbare	J	–	–	T	A	T	TE ins	29-bp ins	14-bp del	T
Tainung 67	J	–	+	T	A	T	TE ins	29-bp ins	14-bp del	G
Nobohai	I	–	+	T	A	T	1-bp del	Both	14-bp del	G
Parahainakoru	I	–	+	T	A	T	Both	Both	14-bp del	G
Ragarasu	I	–	+	T	A	T	1-bp del	Both	14-bp del	G
Tangengenrankatsu	I	–	++	T	A	T	1-bp del	Both	14-bp del	G
Tapopuri	I	–	++	T	A	T	1-bp del	Both	14-bp del	G
IR64	I	–	+	T	A	T	TE ins	Both	14-bp del	G
Kasalath	I	+++	+++	T	A	T	Both	Both	No deletion	G
Wild rice	–	+++++	+++++	A	G	G	Neither	Neither	No deletion	G

^a^Awn length: ++++, > 10 cm; +++, 5–10 cm; ++, 2–3 cm; −-, awnless

^b^Shattering: the number of 『+』 signs represents the degree of shattering

## Discussion

### The first rices grown in Taiwan were domesticated *japonicas*

We have shown that for most domestication genes, the most primitive Formosan landraces contain the same sequence changes as many known modern cultivars. Both early *japonica* and *indica* accessions have exactly the same haplotypes for the loss-of-function *sh4, prog1* and *lg1* genes. This fits very well with the hypothesis of a single de novo domestication followed by transfer of domestication genes between rice subpopulations through introgression (Choi et al. 2017; Choi and Purugganan 2018). However, it should be noted that there are two haplotypes for each of the two awn-related genes. For *an1,* the early and modern *japonica* accessions have the TE-insertion type and four out of five *indica* accessions have a 1-bp deletion. As to *an2,* 9 out of 10 *japonica* accessions have a 29-bp insertion while all 5 *indica* accessions have both a 29-bp insertion and a 1-bp deletion. To conclude, our study shows that the first rice landraces introduced to Taiwan thousands of years ago were domesticated *japonica* rices. They were neither wild nor domesticated de novo from wild rice.

Our Formosan rice accessions were collected from Austronesian-speaking villages when these populations still lived in considerable isolation from the modern world— for example ritual tooth ablation was still performed in many villages in Taiwan at the end of nineteenth century. Because of several primitive agronomic traits, a recent introduction from the outside is unlikely.

### More genes are responsible for awn length, presence of barbs and shattering in domesticated Rices

Both *an1* and *an2* genes in all 15 accessions tested are loss-of-function. Yet, awn length in these accessions varies from zero to about 5 cm. This indicates that more genes are controlling the presence/absence of an awn as well as its length. Wild rice, including *Oryza rufipogon*, usually has an extra-long awn, much longer than 10 cm; the awn moreover is barbed in wild rice. In contrast, the awns of all aboriginal landraces are barbless. It was noted earlier that loss-of-function *an2* gene leads to a short and barbless awn or to no awn at all (Gu et al. 2015; Hua et al., 2015). Cai and Morishima (2002) showed that awn length in rice is a QTL-controlled trait with more than 10 loci. In addition to *An1* and *An2* used in the current study, *Regulator of Awn Elongation 1* (*RAE1*), *RAE2*, and *RAE3* were shown to contribute to awn length control (Furuta et al. 2015; Bessho-Uehara et al. 2016). Thus, other awn-controlling genes should be responsible for the differences in awn length among Formosan landraces.

Seed shattering was also demonstrated to be a QTL-controlled trait with at least 4 loci (Cai and Morishima 2000). In addition to *sh1* and *sh4* used in the current study, *sh2* (Oba et al. 1995, chr. 1), *sh3* (Eiguchi and Sano 1990, chr. 4) and *sh5* (Cubry et al. 2018, chr. 5) also contribute to the control of shattering. Detailed studies of *sh2* and *sh3* are not available yet. Loss-of-function *sh5* is present mainly in African cultivated rice *Oryza glaberrima* (Cubry et al. 2018). In the present study, all accessions have the same *sh4* haplotype, and all except Nipponbare have the same *Sh1* haplotype. However, the shattering degree of these accessions varies (Table 3 and Additional file 3: Table S1): thus, other genes than *sh1* and *sh4* must be responsible for the observed differences in degree of shattering.

### Models of Taiwan Neolithic origins: CIS vs. NES

The Formosan aboriginal *japonica* rice accessions used in the current study probably all belong to lines ultimately stemming from the center of domestication of *japonica* rice somewhere in the Yangtze basin area. This eliminates a separate event of rice domestication in south China or Taiwan (CIS-1 model) as part of an account of the origin of Austronesian agriculture. The CIS-2 model views agriculture as introduced c. 2800 BCE into Tapenkeng cultures in Taiwan from Neolithic groups “further north or inland”, compatible with a northern domestication of rice. Yet this model also makes the implausible assumption of a sudden and wholesale adoption, by a southern hunter-gatherer group, of a complete northern Chinese Neolithic package including domesticated cereals (foxtail, broomcorn, rice), technologies such as house-building, loom weaving, net fishing, and cultural traits (ritual tooth ablation, sacred foxtail) without offering a mechanism for intimate contact with northern populations. The CIS-2 model further fails to provide any kind of explanation for the Y-chromosome, mtDNA and tooth ablation evidence (below), which implies a southward coastal expansion from Shandong. It also does not account for marked differences in food procurement strategies between pre-agricultural Tapenkeng in Taiwan and contemporary hunter-gatherer sites in coastal south China: at about 3000 BCE, Tapenkeng culture relied primarily on fishing and intertidal foraging, whereas the hunter-gatherer sites across the straits exploited sago palms, bananas, freshwater roots and tubers, fern roots, acorns, Job's-tears as well as wild rice, with sago palms having particular importance (Yang et al. 2013): these elements are not prominent in pre-agricultural Tapenkeng sites in Taiwan. Pre-agricultural ceramic sites in late 4th and early 3rd millennium BCE Taiwan are better viewed as temporary or seasonal settlements by Austronesian fishermen and foragers who had preceded Austronesian farmers on the island. Agriculture is the responsibility of women among modern Formosan groups, whereas men engage in fishing, long-distance expeditions and warfare (Adelaar 2012). The Austronesian move to Taiwan may have been initiated through fishing and/or foraging expeditions by pre-Austronesian men from the Fujian coast while the women, and farming, waited on the other side.

In a recent development within the CIS-2 model, (Deng et al. 2017) argue for a spread of millet to Taiwan along an inland route originating in Anhui or Hunan and passing through Jiangxi and Fujian. They note the presence of foxtail c. 3800 BCE at Chengtoushan in Hunan (mid-Yangtze Valley); they themselves discovered foxtail, boomcorn and rice cultivated together in two coastal north Fujian sites at 2000–1500 BCE. However, foxtail at Chengtoushan was a minor cereal introduced from the north into a long-established Yangtze Valley rice tradition. It would be very difficult on this basis to explain the sacred status of foxtail among the Austronesians of Taiwan. The two Fujian sites with foxtail are moreover too late to constitute traces of a spread of agriculture to Taiwan before 2800 BCE. The presence of the three cereals at these sites is actually perfectly consistent with our NES hypothesis of a southward spread of the foxtail-broomcorn-rice trio along a coastal route. The inland route hypothesis also has to explain why broomcorn has never been observed archaeologically in south China before the earliest Formosan agriculture. (Deng et al. 2017) do not actually exclude an expansion of northern agriculture out of Shandong along a coastal route, as under the NES model.

### Models of Taiwan Neolithic origins: LY vs. NES

The remaining NES and LY models both involve an introduction from the outside of already domesticated rice to Taiwan by the first Austronesians. Rice was much less prominent than the millets in the NES region but its presence alongside millet is continuous from Houli culture at 6000–5500 BCE in north Shandong (Crawford et al. 2006; Jin et al. 2014) to south-central Shandong c. 5000 BCE (Yuhuanding, phytoliths: Jin et al. (2010)) to Dongpan at 4030–3820 BCE in southern Shandong (Wang et al. 2012). d'Alpoim Guedes et al. (2015) show that north Shandong was ecologically suitable for rice cultivation in the climatic optimum period 6000–5000 BCE. A southward shift of the northern limit of rice cultivation at the end of that period accords with expectations.

**Fig. 4:**
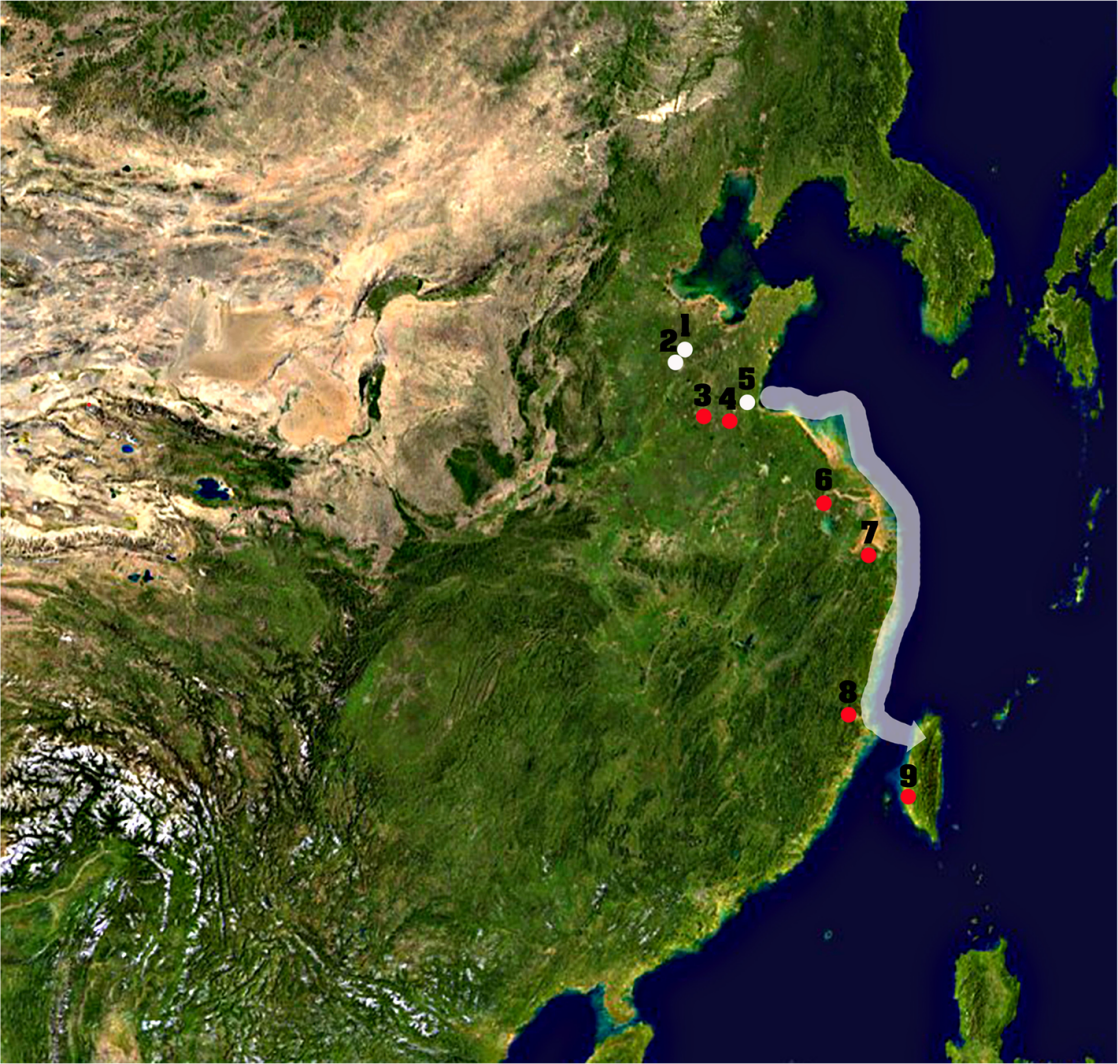
Archaeological sites in this study and the proposed migration route. 1, Zhangmatun; 2, Yuezhuang; 3, Beixin; 4, Dadunzi; 5, Dongpan; 6, Weidun; 7, Hemudu; 8, Tanshishan; 9, Nankuanli. Sites where tooth ablation is reported are indicated by red dots. The arrow shows the proposed migration route of the pre-Austronesians from Shandong to Taiwan. The northeastern Asia image was downloaded from https://commons.wikimedia.org/wiki/File:Asia_satellite_orthographic.jpg originally from NASA

Anthropological and genetic evidence can be cited in support of the NES model. The custom of ritual ablation of the upper maxillary incisors in boys and girls first appears in the Beixin culture of Shandong c. 5000 BCE. The main authors on neolithic tooth ablation: (Han and Nakahashi 1996; Yang 2005) point out a southward expansion of the custom, with younger dates as tooth ablation moves south: the custom reached the north of the Yangtze delta c. 4510 BCE at Dadunzi; Weidun in the lower Yangtze in 4170–3270 BCE (see Han and Nakahashi 1996:45 for dates and details); after 3000 BCE Tanshishan in the Fuzhou basin (Lauer et al. 2012) and Nan Kuan Li on the west coast of Taiwan c. 2800 BCE (Pietrusewsky et al. 2014). The gradual southward spread of tooth ablation from Shandong to Taiwan (Fig. 4) can serve as a geographical and temporal marker of the southward progress of the millet- and rice-cultivating pre-Austronesians along the China coast. The geography of two unilaterally-inherited Austronesian genetic markers—the mtDNA E haplogroup and the Y-chromosome O3a2b2-N6 haplogroup—is consistent with our demic expansion scenario. Precursors of these markers concentrate in coastal regions north of Fujian (Ko et al. 2014; Wei et al. 2017), along the proposed expansion route. Both markers were further shown to have close ties to corresponding markers among Sino-Tibetan populations, which originate in the Yellow River Valley. Thus the mtDNA E haplogroup originates in the M9 haplogroup, whose sister the M9a haplogroup is largely limited to Sino-Tibetan populations (Ko et al. 2014, Wei et al. 2017). The date of separation between M9 and M9a has been placed in the period 6000–8000 BCE (Ko et al. 2014).

The evidence is much less supportive of the LY model. The rice-cultivating cultures of the lower Yangtze such as Hemudu have neither tooth ablation nor any one of the two millets. Rice was grown but there are clear differences in the degree of domestication, specialization and in cultivation techniques. Rice grain sizes are larger in the Lower Yangtze/Hangzhou Bay area than in early Shandong and Taiwan (Fuller 2011). Rice was the only cereal in the Lower Yangtze/Hangzhou Bay area, whereas in Shandong and Taiwan, millets were more prominent. Permanent fields with water management in Hangzhou Bay area sites (Fuller and Qin 2009) are without equivalent in Taiwan or in Shandong, where in contrast, the absence of any traces of permanent fields makes cultivation without water management likely for all three cereals. If the Formosan Neolithic were an offshoot of the Hangzhou Bay area Neolithic, one would expect to find permanent fields and water management in Taiwan and, after nearly two additional millennia of domestication, larger rice grains in Taiwan than in the Hangzhou Bay area. One would also expect to find seeds of paddy field weeds such as *Echinochloa crus-galli.* Until the twentieth century, Formosan rice was cultivated in non-irrigated upland fields, like the millets. Upland fields, whether for rice, foxtail or broomcorn, are referred to in Formosan languages by means of an indigenous word, often a reflex of Proto-Austronesian *qumah. Irrigated paddy rice cultivation was introduced by Chinese settlers in the past centuries (Imbault-Huart 1893): accordingly there is no old Austronesian word for the irrigated rice field in Taiwan or outside of Taiwan.

Following the promotion of irrigated rice cultivation during the Japanese occupation (1895–1945) (Iso 1944), paddy rice has grown in economic importance during the twentieth century, but many older Austronesian speakers remember that foxtail, rather than paddy rice, was the staple still in the middle of the twentieth century (Namoh 2013). The sacred character of foxtail and its recent status as the staple food of Formosan Austronesians strongly indicate that foxtail was culturally more central than rice and broomcorn to the early Austronesians. This argues against the LY model. Because foxtail millet has great antiquity in northeast China, it supports the NES model.

### Comparing plant materials from Shandong, lower Yangtze and Taiwan neolithic sites

To further illustrate the differences between the NES and LY Neolithic, we compare the domesticated and non-domesticated plants found in Shandong, Taiwan and Hangzhou Bay area neolithic sites (Table 4). Foxtail millet and broomcorn millet were present in both Shandong and Taiwan but have not been found in Lower Yangtze/Hangzhou Bay sites. Aquatic nuts (*Trapa* spp., *Euryale ferox*) formed an important part of the subsistence in the Hangzhou Bay Neolithic (Deng et al. 2015) but are virtually unknown in early Neolithic sites in Taiwan and are rare in the Shandong Houli and Beixin/Dawenkou cultures. Wild barnyard grasses (*Echinochloa* spp.) were harvested and consumed before 5000 BCE in the Hangzhou Bay area (Yang et al. 2015) but have not been reported as a significant source of food in either Taiwan or the Houli and Beixin/Dawenkou cultures of Shandong. Finally, ritual tooth ablation, present in the Houli and Beixin/Dawenkou cultures of Shandong, in the early Formosan Neolithic and in scattered locations between Shandong and Taiwan, has not been reported in the main Hangzhou Bay sites.

**Table 4:** The principal plant foods in three Neolithic regions on the China coast. Three domesticated plants (rice, foxtail millet, broomcorn millet) and three non-domesticated ones (water chestnuts, foxnuts, barnyard grasses) are listed

Plants	Excavated region		
	Shandong	Taiwan	Hangzhou Bay area
Rice	+	+	+
Foxtail millet	+	+	–
Broomcorn millet	+	+	–
Water chestnuts (*Trapa* spp.)	–	–	+
Foxnuts (*Euryale ferox*)	(−)	–	+
Barnyard grasses	–	–	+

### The hypothesis of a Shandong origin of the Formosan neolithic

To recapitulate, the presence in Shandong well before the onset of the Formosan neolithic of an agricultural system associating foxtail, broomcorn and small quantities of rice, accompanied by ritual tooth ablation, make Shandong the stronger candidate precursor of the Formosan Neolithic (Ko et al. 2014; Sagart 1995; Fuller et al. 2010; Stevens et al. 2016; Sagart 2008).

The population expansion signal detected at c. 6000–8000 BCE in the Austronesian mtDNA E haplogroup by geneticists (Ko et al. 2014) may represent millet-fueled population growth c. 8000 BCE preceding and during the early Houli culture, followed at c. 6000 BCE by the addition of rice to the original repertoire. Population growth stimulated by diversified cereal agriculture led groups in north Shandong to expand south (since during the climatic optimum, Shandong was the northern limit of rice cultivation) shortly afterwards, their expansion materialized by the southward progress of tooth ablation. We suggest that in the late 4th millennium BCE, these groups, some of whose members carried the mtDNA M9E haplogroup and/or the Y chromosome O3a2b2-N6 haplogroup, introduced to Taiwan the Proto-Austronesian language; a mixed farming, fishing and intertidal foraging subsistence strategy; domesticated landraces of foxtail millet, broomcorn millet and *japonica* rice; a belief in the sacredness of foxtail millet; ritual ablation of the upper incisors in adolescents of both sexes; domesticated dogs; and a technological package including inter alia houses, nautical technology, and loom weaving. Better than other models, the hypothesis of a southward demic expansion out of Shandong provides a credible account of the Austronesian settlement of Taiwan.

## Conclusion

Our botanically informed linguistic fieldwork converges with earlier findings in archaeology and genetics to assign a lesser role for rice than was earlier thought, and a more important one for the millets. Our study of domestication genes in a collection of traditional rice landraces maintained by the Formosan aborigines shows that early Taiwanese rices were introduced to the island in already domesticated form. We argue that domesticated rice and millets were brought to Taiwan by a population having expanded south along the coast from Shandong after c. 5000 BCE, reaching western Taiwan in the second half of the 4th millennium BCE.

## Methods

### Linguistic fieldwork

In the summer and fall of 2017, we visited 16 Taiwanese villages where the Formosan languages Amis, Atayal, Bunun, Kanakanabu, Kavalan, Kaxabu, Paiwan, Rukai, Saaroa, Saisiyat, Sediq and Thao are spoken (Additional file 3: Table S3). There we collected lexical data relevant to eight words reconstructed at the earliest level (Proto-Austronesian) in an online reference work on the Austronesian vocabulary (Blust and Trussel 2016), all of them with attributed rice-specific meanings: *Semay “cooked rice”, *beRas “dehusked rice”, *qeCah “rice husk/bran”, *bunabun “rice seedling”, *bineSiq “seed rice”, *qemu “sticky rice cake”, *bayu “to pound rice”, *iŋsuŋ “rice mortar”. The data were collected as part of a larger survey of the Formosan vocabulary of traditional agriculture. The survey team included the second and third authors, YCT and TFH, two botanists, and the first author, LS, a linguist. Informants from villages where millet agriculture had been reported were selected for both proficiency in the language and experience in agriculture. They were informed of the survey's aims and signed informed consent sheets. Most informants were elderly. In practice, except in protected mountain areas, younger speakers are not proficient enough and/or do not have direct experience with millet cultivation. Interviews were conducted in the informants' homes and/or fields. Questions were formulated in Mandarin Chinese, with the aid of samples and pictures of plants or by pointing at objects of interest when these were present in the environment: when an informant did not know Chinese, a local bilingual speaker translated the question into the informant's native language. Responses were interpreted back and forth and transcribed into IPA by LS. We aimed at a systematic phonetic transcription rather than a narrow phonetic one.

### Selection of rice landrace accessions

Out of our collection of 60 aboriginal landraces from Taiwan, we selected 15 for whole-genome sequencing and follow-up analysis, taking care to include accessions with primitive traits such as red pericarp, extra-long awn (around 5 cm) and shattering. The domestication-related traits of these 15 Formosa rice accessions, plus Kasalath (a primitive Aus rice from Bangladesh), Tainung 67 (TNG67, a modern Taiwanese *japonica* variety), and Nipponbare (a Japanese modern *japonica* variety) are shown in Additional file 3: Table S4. To our original 60 upland accessions, we added Nipponbare, IR64 (a modern *indica* variety), Pai-K'o-Tsao-Tzu and O-Loan-Chu (two *indica* landraces grown in Taiwan since the eighteenth century): these 64 landraces were then subjected to STRUCTURE analysis (Fig. 2). In a further phylogenetic comparison, we used the genome sequence data from 55 accessions: the set of 15 Formosan landraces described above, plus 40, consisting of: 10 Asian AA genome wild rice, including 5 *Oryza nivara* and 5 *O. rufipogon*; 7 temperate *japonica*; 6 tropical *japonica*; 7 *indica*, 4 *Aus*, and 6 aromatic. Each subtype contained landraces and modern varieties. The sequence data for these accessions were gathered from Xu et al. (2012), our previous work (Wei et al. 2016a; Wei et al. 2016b) and from results obtained for this study. The names, types, origins, and DNA accession numbers are shown in Additional file 3: Table S4.

### Identification of the subtypes of landraces and cultivars

The chloroplast DNA for *japonica* and *indica* has minor differences. For instance, the open reading frame 100 (ORF100) is 23 amino acid residues less for *indica* than *japonica* rice (Kanno et al. 1993). Thus, the ORF100 polymerase chain reaction (PCR) product is 69-bp less for *indica* than for *japonica*. By the method retrotransposon-based insertion polymorphism (RBIP) of Panaud and colleagues (Vitte et al. 2004), the PCR product is about 100-bp higher for *japonica* than for *indica*. The primer sequences for both methods are shown in Additional file 3: Table S5.

### Whole-genome sequencing and data interpretation

Genomic DNA from rice plants was extracted from healthy leaves of a single-seed–descent plant by using the DNeasy Plant Mini Kit (Qiagen). After quality assessment, genomic DNA was randomly fragmented and size-fractionated. DNA fragments with the desired lengths were gel-purified. For whole-genome resequencing, paired-end libraries with 450- to 500-bp inserts were constructed and sequenced by using a GA2 or HiSeq2000 system (Illumina). Adaptor sequences, low-quality bases and reads < 20-bp long were discarded. The trimmed paired reads were aligned to the reference rice Nipponbare genome sequence (IRGSP v1.0) (Project, 2005, Kawahara et al. 2013). SAMtools and VCFtools (Danecek et al. 2011a; Li et al. 2009) were used to manipulate and transform the sequence alignment/map format (SAM) and variant call format (VCF) (Danecek et al. 2011b) of the file. To detect SNPs and small indels, we used the command lines in the section “EXAMPLES” in the SAMtools manual without any restriction on depth or mapping quality. The information on single nucleotide polymorphisms (SNPs) and small insertions/deletions (indels) was recorded in VCF files. The sequence data for all landraces were deposited into the NCBI Sequence Read Archive.

### STRUCTURE analysis

We used simple sequence repeat (SSR) markers and target induced local lesions in genomes (TILLING) (McCallum et al. 2000) results for several domestication-related genes, including *Headingdate1* (*Hd1*) (Yano et al. 2000), *Headingdate 3a* (*Hd3a*) (Monna et al. 2002), *Headingdate 6* (*Hd6*) (Yamamoto et al. 2000)*, Early headingdate 1* (*Ehd1*) (Doi et al. 2004), *Early headingdate 2* (*Ehd2*) (Matsubara et al. 2008), *Photoperiodic sensitivity 5* (*SE5*) (Izawa et al. 2000), and *Waxy* (Wang et al. 1995). We also sequenced the functional SNP of *QTL for rice seed width on chromosome 5* (*qSW5*) (Shomura et al. 2008)*,* aroma rice gene *BADH1* (Bradbury et al. 2005)*,* seed shattering gene *qSh1* (Konishi et al. 2006)*, Grain size 3* (*GS3*) (Fan et al. 2006)*, Grain width 2* (*Gw2*) (Song et al. 2007)*,* seed dormancy *Sdr4* (Sugimoto et al. 2010)*,* and red caryopsis gene *red caryopsis* (*Rc*) (Sweeney et al. 2006). To reveal the population structure of the 60 Formosan rice accessions, we used 344 alleles, including SSR markers, TILLING and sequencing results, with the model-base program STRUCTURE (Pritchard et al. 2000) and to identify the proper number of populations (K). Three independent runs were performed for each simulated value of K, ranging from 1 to 5. The primer sequences used are in Additional file 3: Table S5.

### Phylogenetic analysis

To reveal the position of the Formosan rice accessions relative to other Asian rice, including five cultivated subtypes and two wild rice species, we performed a phylogenetic analysis with next-generation sequencing (NGS) data. Our 15 primitive Formosan accessions plus 40 accessions, including wild rice and five cultivated rice subgroups, were used in the phylogeny analysis. Additional file 3: Table S4 lists the names, types, origins and sequence information for these lines. The clean reads were mapped to the Nipponbare reference genome (IRGSP v1.0) by using BWA v0.7.13-r1126 mem with default parameters (Li and Durbin 2010; Kawahara et al. 2013). The mapped results were merged and low mapping quality (q < 20) data were removed as BAM files by using Samtools v1.3 (Li et al. 2009; Li 2011). Picard v2.1.1 MarkDuplicates was used to identify and remove duplicate reads originating in the same DNA fragments (http://broadinstitute.github.io/picard/). The Genome Analysis Toolkit v3.5–0-g36282e4 RealignerTargetCreator was used to identify regions around indels, then the Genome Analysis Toolkit IndelRealigner was used to execute local realignment (McKenna et al. 2010). Samtools and Bcftools were used to call for variant calling including SNPs and indels with filter by depth and mapping quality. Genetic distance with the p-distances model was calculated, and a neighbor-joining tree was constructed with 1000 bootstraps by using PHYLIP v3.695 (http://evolution.genetics.washington.edu/phylip.html). MEGA v7 (Kumar et al. 2016) was used to display the phylogenetic tree.

## Additional files

Additional file 1: **Figure S1.** The Austronesian language family. Source: The Language Gulper (http://www.languagesgulper.com/eng/Austronesian.html). (TIF 16508 kb)

Additional file 2: **Figure S2.** Morphology of seed and caryopsis of all 60 aboriginal landraces. N, Nipponbare; I, IR64; 1, Pai-Ko-Tsao-Tzu; 2, O-Loan-Chu; 3, Purahaitairin; 4, Hopots utaiyaru; 5, Ragasu; 6, Pairauwar; 7, Nutsurikui; 8, Midon; 9, Burieuraozu; 10, Papito; 11, Mandarakiku; 12, Pairaur; 13, Tangengenrankatsu; 14, Paotsupagaiahon; 15, Montana; 16, Muteka; 17, Haifugoya; 18, Nata-ra; 19, Pazumatamaru; 20, Kabofu; 21, Tahobin; 22, Nabohai; 23, Nakairitsu; 24, Munagurusu; 25, Kabotsumame; 26, Bohai; 27, Gurusu; 28, Matara; 29, Nobohai; 30, Parahainakoru; 31, Habun No.1; 32, Nakarofukarapai S1; 33, Napatsupai; 34, Chuan No. 2; 35, Chuan No. 3; 36, Chuan No. 4; 37, Ragarasu; 38, Tapopuri; 39, unknown; 40, Pakaikauneku; 41, Kaisentetsuchitsu; 42, Napatsupai S3; 43, Baridon; 44, Paerizumochi; 45, Tarunatsumochi; 46, Warisanmochi 1; 47, Warisanmochi 2; 48, Szu Ming Lu Tao; 49, Komapatai; 50, Pagaitsuitaiyaru; 51, Airaromu; 52, Pazumataharu; 53, Nakara 2; 54, Nakabo; 55, Naguton; 56, Komonawai; 57, Pintowan 1; 58, Koodngoi; 59, Kahorui; 60, Tongsisai; 61, Banadoion; 62, Patsupatsu. (TIF 1961 kb)

Additional file 3: **Table S1.** Aboriginal rice accessions, control varieties and their domestication-related phenotypes. **Table S2.** Information on functionally characterized genes and mutations that underlie phenotypic changes during rice domestication. **Table S3.** Tribe, village and gender of informants. **Table S4.** Accessions used in the phylogenetic study, regions collected and their DNA accession numbers. **Table S5.** Primers used in the studies. (DOCX 67 kb)

## Abbreviations

CIS: Chinese Interaction Sphere
indels: insertions/deletions
LY: Lower Yangtze
NES: Northeastern Seaboard
NGS: next-generation sequencing
NKLE: Nan Kuan Li East
ORF: open reading frame
RBIP: retrotransposon-based insertion polymorphism
SAM: sequence alignment/map format
SNP: single nucleotide polymorphisms
SSR: simple sequence repeat
TILLING: target induced local lesions in genomes
TNG67: Tainung 67
VCF: variant call format
