# Supplementary figures and images for: A northern Chinese origin of Austronesian agriculture: new evidence on traditional Formosan cereals
Source: Rice (N Y). 2018 Oct 11;11:57. doi: 10.1186/s12284-018-0247-9 (PMC6179969; doi:10.1186/s12284-018-0247-9)

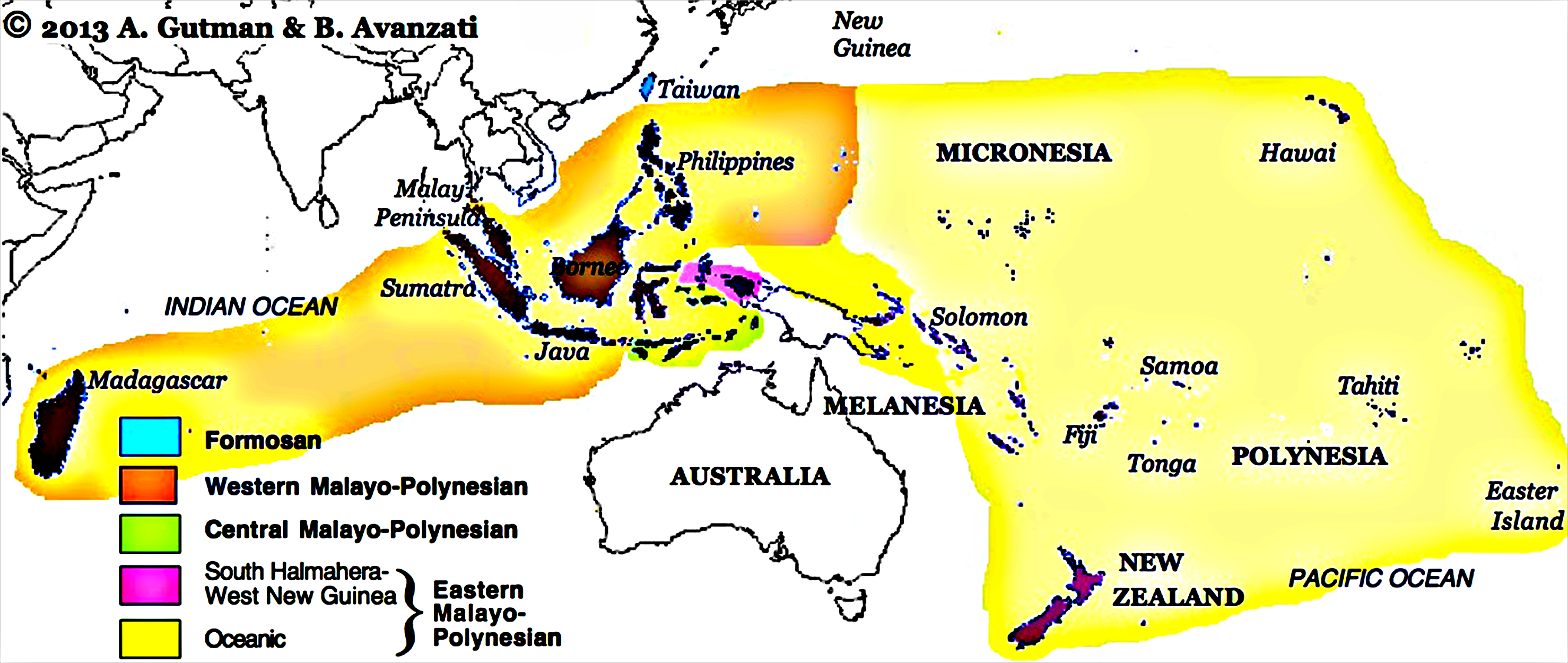

Supplement: Supplementary file 1 — Figure S1. The Austronesian language family. Source: The Language Gulper (http://www.languagesgulper.com/eng/Austronesian.html). (TIF 16508 kb) [file 12284_2018_247_MOESM1_ESM.tif]

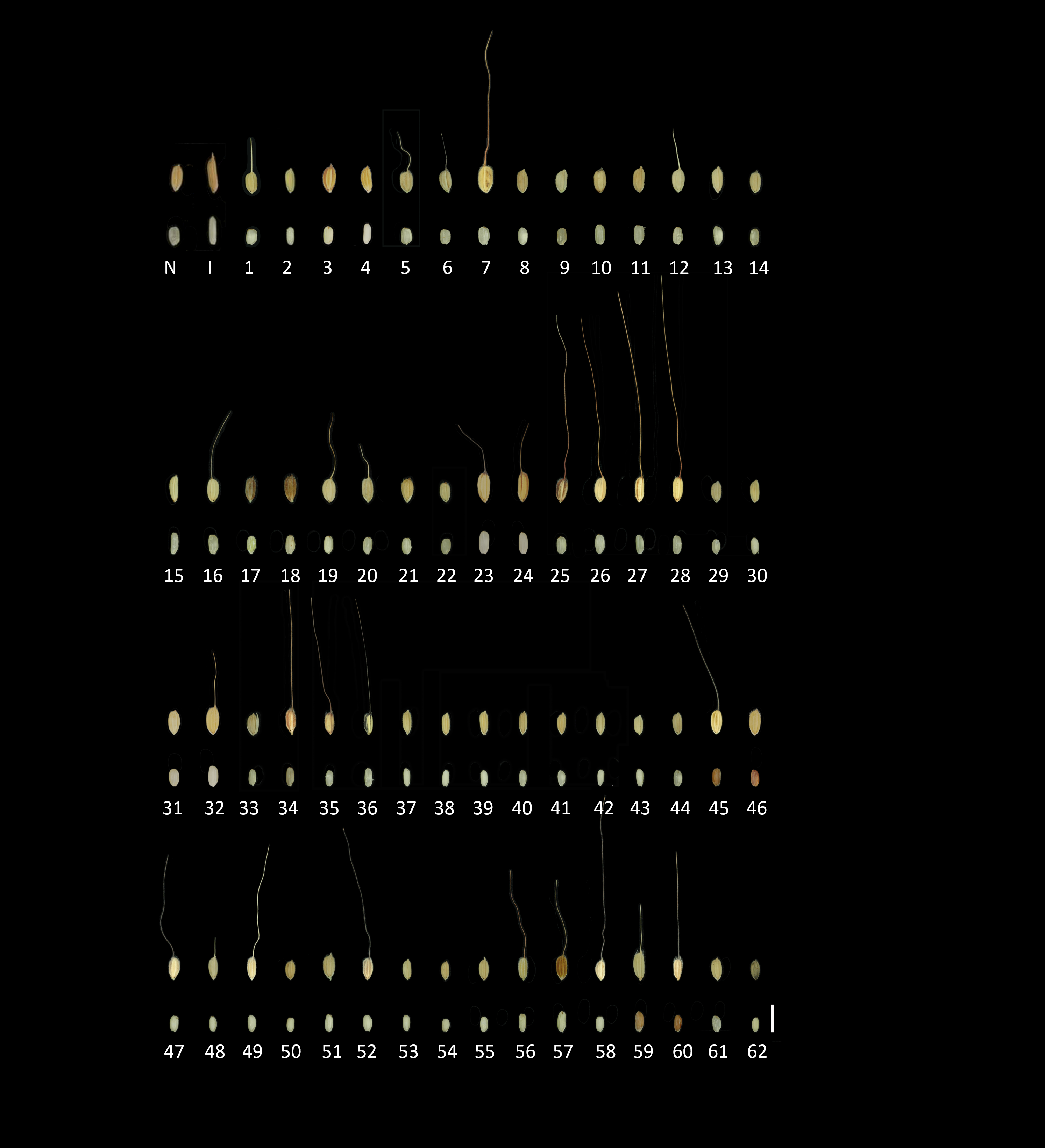

Supplement: Supplementary file 2 — Figure S2. Morphology of seed and caryopsis of all 60 aboriginal landraces. N, Nipponbare; I, IR64; 1, Pai-Ko-Tsao-Tzu; 2, O-Loan-Chu; 3, Purahaitairin; 4, Hopots utaiyaru; 5, Ragasu; 6, Pairauwar; 7, Nutsurikui; 8, Midon; 9, Burieuraozu; 10, Papito; 11, Mandarakiku; 12, Pairaur; 13, Tangengenrankatsu; 14, Paotsupagaiahon; 15, Montana; 16, Muteka; 17, Haifugoya; 18, Nata-ra; 19, Pazumatamaru; 20, Kabofu; 21, Tahobin; 22, Nabohai; 23, Nakairitsu; 24, Munagurusu; 25, Kabotsumame; 26, Bohai; 27, Gurusu; 28, Matara; 29, Nobohai; 30, Parahainakoru; 31, Habun No.1; 32, Nakarofukarapai S1; 33, Napatsupai; 34, Chuan No. 2; 35, Chuan No. 3; 36, Chuan No. 4; 37, Ragarasu; 38, Tapopuri; 39, unknown; 40, Pakaikauneku; 41, Kaisentetsuchitsu; 42, Napatsupai S3; 43, Baridon; 44, Paerizumochi; 45, Tarunatsumochi; 46, Warisanmochi 1; 47, Warisanmochi 2; 48, Szu Ming Lu Tao; 49, Komapatai; 50, Pagaitsuitaiyaru; 51, Airaromu; 52, Pazumataharu; 53, Nakara 2; 54, Nakabo; 55, Naguton; 56, Komonawai; 57, Pintowan 1; 58, Koodngoi; 59, Kahorui; 60, Tongsisai; 61, Banadoion; 62, Patsupatsu. (TIF 1961 kb) [file 12284_2018_247_MOESM2_ESM.tif]
