# Supplementary material for: A northern Chinese origin of Austronesian agriculture: new evidence on traditional Formosan cereals
Source: Rice (N Y). 2018 Oct 11;11:57. doi: 10.1186/s12284-018-0247-9 (PMC6179969; doi:10.1186/s12284-018-0247-9)
Supplement: Supplementary file 3 — Table S1. Aboriginal rice accessions, control varieties and their domestication-related phenotypes. Table S2. Information on functionally characterized genes and mutations that underlie phenotypic changes during rice domestication. Table S3. Tribe, village and gender of informants. Table S4. Accessions used in the phylogenetic study, regions collected and their DNA accession numbers. Table S5. Primers used in the studies. (DOCX 67 kb) [file 12284_2018_247_MOESM3_ESM.docx]

Additional file 1

**Table S1. The aboriginal rice accessions, the control varieties and their domestication-related phenotypes**

|  | **Type** | **Sub-**  **spp** | **Awn length**^a^ | **Shattering degree**^b^ | **Plant stature** | **Panicle phenotype** | **Caropsis color** |
| --- | --- | --- | --- | --- | --- | --- | --- |
| **Bohai** | Aboriginal | J | +++ | +++ | Erect | Closed | White |
| **Chuan No4** | Aboriginal | J | +++ | +++ | Erect | Closed | White |
| **Kabotsumame** | Aboriginal | J | +++ | +++ | Erect | Closed | White |
| **Matara** | Aboriginal | J | ++ | +++ | Erect | Closed | White |
| **Montana** | Aboriginal | J | - | +++ | Erect | Closed | White |
| **Muteka** | Aboriginal | J | ++ | ++ | Erect | Closed | White |
| **Nakabo** | Aboriginal | J | - | ++ | Erect | Closed | White |
| **Nakairitsu** | Aboriginal | J | ++ | + | Erect | Closed | White |
| **Purahaitairin** | Aboriginal | J | + | + | Erect | Closed | White |
| **RuiYan** | Aboriginal | J | ++ | ++ | Erect | Closed | Red |
| **Nipponbare** | Modern | J | - | - | Erect | Closed | White |
| **Tainung 67** | Modern | J | - | + | Erect | Closed | White |
| **Nobohai** | Aboriginal | I | - | + | Erect | Closed | White |
| **Parahainakoru** | Aboriginal | I | - | + | Erect | Closed | White |
| **Ragarasu** | Aboriginal | I | - | + | Erect | Closed | White |
| **Tangengenrankatsu** | Aboriginal | I | - | ++ | Erect | Closed | White |
| **Tapopuri** | Aboriginal | I | - | ++ | Erect | Closed | White |
| **IR64** | Modern | I | - | + | Erect | Closed | White |
| **Kasalath** | Land race | I | +++ | +++ | Erect | Closed | Red |
| **Wild rice**^c^ | Wild rice | -- | +++++ | +++++ | Open | Open | Red |

^a^ Awn length: ++++, >10 cm; +++, 5-10 cm; ++, 2-3 cm; --, awnless.

^b^ Shattering: the number of ‘+’ signs represents the degree of shattering.

^c^ Both *Oryza nivara* and *O. rufipogon* are included.

**Table S2. Information on functionally characterized genes and mutations that underlie phenotypic changes during rice domestication.**

| Gene symbol | Gene locus | Phenotype in wild rice | Phenotype in the domesticated rice | Molecular function | Nucleotide changes | Protein changes | Reference |
| --- | --- | --- | --- | --- | --- | --- | --- |
| *An1* | Os04g0350700 | Long awn | Decrease in awn length | a basic helix-loop-helix (bHLH) protein | A 1-bp deletion or a transposon-like (TE) insertion | Frameshift in the open reading frame (ORF), loss of function | (Luo et al, 2013) |
| *An2 (LABA1)* | Os04g0518800 | Long and barbed awn | Decrease in awn length and barbless | Lonely Guy like protein (*OsLOGL6*) | 1-bp deletion (C) or 29-bp insertion | Frameshift in the ORF, loss of function | (Gu et al, 2015, Hua et al, 2015) |
| *Sh1* | Os01g0848400 | Shattering seed | Less shattering seed | BEL1-type homeobox family | G to T | Change in the regulatory region | (Konishi et al, 2006) |
| *Sh4* | Os04g0670900 | Shattering seed | Less shattering seed | Homeodomain-like containing protein | G to T | Alteration in the ORF | (Li et al, 2006) |
| *PROG1* | Os07g0153600 | Spreading tiller | Erect plant architecture | A zinc-finger transcription factor | A to T | Alteration in the ORF | (Jin et al, 2008) |
| *OsLG1* | Os04g0656500 | Spread panicle | Closed-panicle architecture | A SQUAMOSA promoter-binding protein | G to A | Change in the regulatory region | (Ishii et al, 2013, Zhu et al, 2013) |
| *Rc* | Os07g0211500 | Reddish-brown caryopsis | White caryopsis | A bHLH protein | A 14-bp deletion | Frameshift in the ORF, loss of function | (Sweeney et al, 2006) |

**Table S3.** **Tribe, village and gender of informants.**

| **Tribe** | **Village** | **Gender** |
| --- | --- | --- |
| Amis | Dulan | F |
| Amis | Fata'an | M |
| Atayal | Jianshih | F |
| Bunun | Shuanglong | M |
| KanaKanabu | Namasia | F,M |
| Kavalan | Xinshe | M |
| Kaxabu | Puli | M |
| Paiwan | Dewen | F |
| Paiwan | Daniao | M |
| Paiwan | Taiban | F,M |
| Rukai | Dewen | M |
| Rukai | Taromak | F |
| Saisiyat | Nanjhuang | M |
| Sediq | Cingliou | F |
| Thao | Dehau | F |

**Table S4. Accessions used in the phylogenetic study, regions collected and their DNA accession numbers.**

| **ID** | **Name** | **Species** | **type** | **Origin** | **DNA accessions** | **References** |
| --- | --- | --- | --- | --- | --- | --- |
| IRGC12793 | Kitrana 508 | *O. sativa* | Aromatic | Madagascar | SRR063606 | (Xu et al, 2012) |
| IRGC38994 | Bico Branco | *O. sativa* | Aromatic | Brazil | SRR063605 | (Xu et al, 2012) |
| IRGC9060 | JC101 | *O. sativa* | Aromatic | India | SRR063612 | (Xu et al, 2012) |
| IRGC9062 | JC111 | *O. sativa* | Aromatic | India | SRR063594 | (Xu et al, 2012) |
| IRGC31856 | KUI SALI | *O. sativa* | Aromatic | Bangladesh | SRR063618 | (Xu et al, 2012) |
| RA4952 | Firooz | *O. sativa* | Aromatic | Iran | SRR063602 | (Xu et al, 2012) |
| IRGC45975 | Kalamkati | *O. sativa* | AUS | India | SRR063604 | (Xu et al, 2012) |
| IRGC6307 | Jhona 349 | *O. sativa* | AUS | India | SRR063592 | (Xu et al, 2012) |
| IRGC8555 | DZ78 | *O. sativa* | AUS | Bangladesh | SRR063599 | (Xu et al, 2012) |
| Kasalath | Kasalath | *O. sativa* | AUS | Bangladesh | ERS470551 | NCBI |
| IRGC9148 | TD2 | *O. sativa* | Indica | Thailand | SRR063593 | (Xu et al, 2012) |
| IRGC51300 | Guan-Yin-Tsan | *O. sativa* | Indica | China | SRR063628 | (Xu et al, 2012) |
| fluffy | San-Pei | *O. sativa* | Indica | Taiwan | SRR5239877 | Current study |
| EF1 | Shuang-Chiang-Tsao-1 | *O. sativa* | Indica | Taiwan | SRR5239948 | Current study |
| TCS17 | Taichung Sen 17 | *O. sativa* | Indica | Taiwan | SRR5242461 | Current study |
| TNGS20 | Tainung Sen 20 | *O. sativa* | Indica | Taiwan | SRR5242463 | Current study |
| UL34 | Tangengenrankatsu | *O. sativa* | Indica | Taiwan | SRR7691064 | Current study |
| UL50 | Nobohai | *O. sativa* | Indica | Taiwan | SRR7691068 | Current study |
| UL51 | Parahainakoru | *O. sativa* | Indica | Taiwan | SRR7691060 | Current study |
| UL58 | Ragarasu | *O. sativa* | Indica | Taiwan | SRR7691069 | Current study |
| UL59 | Tapopuri | *O. sativa* | Indica | Taiwan | SRR7691067 | Current study |
| IR64 | IR64 | *O. sativa* | Indica | Philippines | SRR3098100 | NCBI |
| RuiYan | Rui Yan Shiang Mi | *O. sativa* | Japonica | Taiwan | SRR5239949 | Current study |
| TNG72 | Tainung 72 | *O. sativa* | Japonica | Taiwan | SRR5242462 | Current study |
| UL37 | Muteka | *O. sativa* | Japonica | Taiwan | SRR7691065 | Current study |
| UL44 | Nakairitsu | *O. sativa* | Japonica | Taiwan | SRR5239878 | Current study |
| UL46 | Kabotsumame | *O. sativa* | Japonica | Taiwan | SRR7691063 | Current study |
| UL47 | Bohai | *O. sativa* | Japonica | Taiwan | SRR7691062 | Current study |
| UL49 | Matara | *O. sativa* | Japonica | Taiwan | SRR5242465 | Current study |
| UL57 | Chuan No4 | *O. sativa* | Japonica | Taiwan | SRR5239629 | Current study |
| TNG67 | Tainung 67 | *O. sativa* | Temperate japonica | Taiwan | SRR1531575 | (Wei et al, 2016a) |
| SR | Shinriki | *O. sativa* | Temperate japonica | Japan | SRR1956772 | (Wei et al, 2016b) |
| KJ | Kameji | *O. sativa* | Temperate japonica | Japan | SRR1956769 | (Wei et al, 2016b) |
| IRGC8191 | Mansaku | *O. sativa* | Temperate japonica | Japan | SRR063630 | (Xu et al, 2012) |
| TC194 | Taichung194 | *O. sativa* | Temperate japonica | Taiwan | SRR5242460 | Current study |
| UL21 | Purahaitairin | *O. sativa* | Temperate japonica | Taiwan | SRR7691061 | Current study |
| UL85 | Nakabo | *O. sativa* | Temperate japonica | Taiwan | SRR7691066 | Current study |
| Nipponbare | Nipponbare | *O. sativa* | Temperate japonica | Taiwan | IRGSP v1.0 | IRGSP |
| IRGC66756 | Lemont | *O. sativa* | Tropical japonica | TX,USA | SRR063613 | (Xu et al, 2012) |
| IRGC17757 | Jambu | *O. sativa* | Tropical japonica | Indonesia | SRR063633 | (Xu et al, 2012) |
| IRGC26872 | Binulawan | *O. sativa* | Tropical japonica | Philippines | SRR063625 | (Xu et al, 2012) |
| IRGC328 | Azucena | *O. sativa* | Tropical japonica | Philippines | SRR063631 | (Xu et al, 2012) |
| IRGC43325 | Arias | *O. sativa* | Tropical japonica | Indonesia (West Java) | SRR063639 | (Xu et al, 2012) |
| IRGC43675 | Trembese | *O. sativa* | Tropical japonica | Indonesia (East Java) | SRR063636 | (Xu et al, 2012) |
| UL36 | Montana | *O. sativa* | Tropical japonica | Taiwan | SRR5242464 | Current study |
| IRGC105327 | Dhoni | *O. nivara* | Wild | Dhoni, India | SRR063622 | (Xu et al, 2012) |
| IRGC106105 | Medinipur | *O. nivara* | Wild | Medinipur, India | SRR063621 | (Xu et al, 2012) |
| IRGC106154 | Vientiane | *O. nivara* | Wild | Vientiane, Laos | SRR063610 | (Xu et al, 2012) |
| IRGC80470 | Madhya Pradesh | *O. nivara* | Wild | Madhya Pradesh, India | SRR063608 | (Xu et al, 2012) |
| IRGC89215 | Sopoir Tep | *O. nivara* | Wild | Sopoir Tep, Cambodia | SRR063611 | (Xu et al, 2012) |
| IRGC105958 | Kromat Watu | *O. rufipogon* | Wild | Kromat Watu, Indonesia | SRR063609 | (Xu et al, 2012) |
| IRGC105960 | Chakaria | *O. rufipogon* | Wild | Chakaria, Bangladesh | SRR063620 | (Xu et al, 2012) |
| P46 | Hainan | *O. rufipogon* | Wild | Hainan, China | SRR063623 | (Xu et al, 2012) |
| VOC4 | VOC4 | *O. rufipogon* | Wild | Nepal | SRR063619 | (Xu et al, 2012) |
| Yuan3-9 | Yunnan | *O. rufipogon* | Wild | Yunnan, China | SRR063624 | (Xu et al, 2012) |

**Table S5. Primers used in the studies.**

**Simple sequence repeat primer sets**

|  | **Forward primer** | **Reverse primer** |
| --- | --- | --- |
| Chromosome 1 | | |
| RM1 | GCGAAAACACAATGCAAAAA | GCGTTGGTTGGACCTGAC |
| S5756 | GTAAAGCATGCGTGCACACC | TTAATAAGAGGCAACGGAAC |
| RM1387 | GTGGCTGGCTGATCGATC | AATCAACCCAGCTACCATGC |
| Chromosome 2 | | |
| CH0226 | TTCTTGTGGGATACAGGAAG | TAAGATCGAGTTGGATGGAG |
| RM341 | CAAGAAACCTCAATCCGAGC | CTCCTCCCGATCCCAATC |
| RM5651 | AAGAGAACATTTGGAATCCT | TAACCTGTGCCTTGTTTG |
| CH0219 | GAATGACCCTAACCTTAGACAA | ATTGGTTATTCCTTGCACAC |
| Chromosome 3 | | |
| RM218 | TGGTCAAACCAAGGTCCTTC | GACATACATTCTACCCCCGG |
| RM251 | GAATGGCAATGGCGCTAG | ATGCGGTTCAAGATTCGATC |
| RM16 | CGCTAGGGCAGCATCTAAA | AACACAGCAGGTACGCGC |
| RM8267 | TGAGGCTGAATAAGATCGAC | TACTCCACTCTCCCGATTTG |
| RM1038 | TGGTTCGATTCGGATTTC | AAGCTATTCACAAGCAGCTC |
| Chromosome 4 | | |
| C61009 | GGCCAGCAAGGTGTAGTAAG | ACAAACCCCAGCACCCTAAG |
| CH0440 | CCTAAGAGTTCAAGGGGAAC | GTCATATTTTCTCCCTGCAT |
| CH0451 | AGAGGAGAACCTTTTCTTGG | ACTGCTTTTGCTACTTTTGG |
| RM252 | TTCGCTGACGTGATAGGTTG | ATGACTTGATCCCGAGAACG |
| RM3648 | TACCCTTTCTTCCCCAAACC | ACCTCCTCCTCCACTTCTCC |
| Chromosome 5 | | |
| RM267 | TGCAGACATAGAGAAGGAAGTG | AGCAACAGCACAACTTGATG |
| RM430 | AAACAACGACGTCCCTGATC | GTGCCTCCGTGGTTATGAAC |
| RM164 | TCTTGCCCGTCACTGCAGATATCC | GCAGCCCTAATGCTACAATTCTTC |
| RM2357 | CCTCCGTTTCACAATGTAAC | CTGATGCTACCAGAATCCTC |
| Chromosome 6 | | |
| SLS163 | TGATGTGATAAAGAAGCAGA | TTTATACTCTTGGTGACGTG |
| RM528 | GGCATCCAATTTTACCCCTC | AAATGGAGCATGGAGGTCAC |
| RM340 | GGTAAATGGACAATCCTATGGC | GACAAATATAAGGGCAGTGTGC |
| RM580 | GATGAACTCGAATTTGCATCC | CACTCCCATGTTTGGCTCC |
| RM6734 | TGAGCAGTCTGCAGATGACC | GCTTGGACTTGGAGTCTTGG |
| Chromosome 7 | | |
| SLS164 | CTGCATATTTTCCCCTATTA | GGACAAGGCACTAATACAGT |
| RM418 | GATCGAGCATCAACACAACG | TTAAGTCTGAAGCCCCTGCT |
| RM234 | ACAGTATCCAAGGCCCTGG | CACGTGAGACAAAGACGGAG |
| CH0701 | CTACTGCTCCGTATTGCTGCT | GTGCCAATTACCTTCCCGTA |
| Chromosome 8 | | |
| RM331 | GAACCAGAGGACAAAAATGC | CATCATACATTTGCAGCCAG |
| CH0877 | AATTTGTAAGTTGCCAAGGA | AATTGGCAAAGAGCTGATTA |
| RM264 | GTTGCGTCCTACTGCTACTTC | GATCCGTGTCGATGATTAGC |
| RM5911 | CCCTCTTTTTAAGTCTGGGG | GGTGCCTCCTTTCAAAGTTG |
| Chromosome 9 | | |
| CH0919 | GTGATCTTCCCCTCGTACTG | GAGGAAGAGTGTGACTCTGAAT |
| RM3912 | TGTGTGTGCCCGATCTAC | CCTCTCGATGAGCATTCC |
| RM278 | GTAGTGAGCCTAACAATAATC | TCAACTCAGCATCTCTGTCC |
| RM6971 | TTTGCGAACTAGACAAGGCC | GCGTCATTCTCGACGAGC |
| Chromosome 10 | | |
| CH1011 | GAAAAGCTTCTTCGTGAATG | CAAAAGCCAAAGAAAGCTAC |
| CH1007 | TTGATCATTGTTTTGCTCTCG | CACCCTGACGCTTAAACTTG |
| C51124 | CACTTCAGTGCTGGGTGTGC | TCAAAGGGCAAGTTAACGAC |
| RM1375 | CTACACGCGCAAACTCTGTC | ATGAAGGTCTAGGCTGCACC |
| CH1016 | AAGCGACAGCGCACGAAAC | GTTATTGGCACGTGGTGTG |
| Chromosome 11 | | |
| RM167 | GATCCAGCGTGAGGAACACGT | AGTCCGACCACAAGGTGCGTTGTC |
| SLS186 | ACGGTAGTAACTGCAAGAAT | CAGGAAATCTGTAACCAGAG |
| RM224 | ATCGATCGATCTTCACGAGG | TGCTATAAAAGGCATTCGGG |
| RM1761 | ACGCTTAAAGAACATTTGAT | GCGATTAACTTTTAACCATT |
| RM1355 | CCTCCACATAAGCGAAGAGC | TGACGCCCAGAAGAGGTATC |
| Chromosome 12 | | |
| RM6905 | GGGACCTGAATTGTCAAATA | AGATAGTAGCTGGGGGTTTC |
| RM247 | TAGTGCCGATCGATGTAACG | CATATGGTTTTGACAAAGCG |
| RM2197 | ACTGAGAACTTTAATCATCG | GAACAACTTTGAAGAGAAAC |

**Primer sequence to differentiate japonica and indica rice**

|  | **Forward primer** | **Reverse primer** |
| --- | --- | --- |
| ORF100 | GTGGACCTGACTCCTTGAA | AGCCGAGGTCGTGGTAA |
| RBIP ( indica) | GTCATCTGGTGGGCGACTATT | AGAACGCAGGCACTCAATCGG |
| RBIP (japonica) | TTCGTAGACACCTGCGAAAAG | AGAACGCAGGCACTCAATCGG |

**Primer sequence for functional single nucleotide polymorphisms in domestication-related genes**

| **gene** | **Forward primer** | **Reverse primer** |
| --- | --- | --- |
| *Badh2* | CCCTGGTGTAGACAAGGTACA | TCCACAGAAATTTGGAAACAA |
| *GS3* | CGTCACTCATTGACCAACATT | TTACTTTCATTTGCCAAGGTTT |
| *Gw2* | GACAACCACTCCTGTCCTGAAATGC | GCACATCGGTATGACGGCACTG |
| *qSH1* | GCATGGATGCGTACAGATT | GCTACTCGCATCCTGTCAAT |
| *qSW5* | GTTGGCATCACTGTTGGAGT | AGCGTGTGTAGGGAAGGAG |
| *Rc* | ACACAAGCACTGCCATTTTT | CCTCTCTTTCAGCACATGGT |
| *Sdr4* | AGAGTGCGGGCGGGGTTAGT | ATCTTGGCCGAGCACGAGAA |
| *Sh-h* | TTTTTGTGCGACAACATCTG | CAGGTCCATTGAAAGAGACG |

**Primer sequences for Targeting Induced Local Lesions in Genomes (TILLING) analysis for domestication-related genes**

| ***Waxy* gene (*Wx*, 9 primer sets)** | |  | |
| --- | --- | --- | --- |
| 1F | CAGCCCAGCTTTCTTCAGCCTGT | | 1R |
| 2F | CTAGCCTCGCCCTGCATGAGAA | | 2R |
| 3F | TGGCTCTGAGGCACTGACGT | | 3R |
| 4F | GCCGAGTTGGTCAAAGGAAAATG | | 4R |
| 5F | AACCACCATGTCGGCTCTCACC | | 5R |
| 6F | CGACCATCCGTCATTCCTGGAG | | 6R |
| 7F | ATGAAGGCCGGAATCCTGGAAG | | 7R |
| 8F | TTCGAGCCCTGTGGACTCATCC | | 8R |
| 9F | CGCCGGCGTACGAGGAGAT | | 9R |
| ***Heading date 1* gene (*Hd1*, 4 primer sets)** | |  | |
| 1F | CTCTACACTCAAACTCCCCAGGAC | | 1R |
| 2F | GACAAGGACGAGGAGGTGGAC | | 2R |
| 3F | GTCAGTGCTTACACAGATTCCATC | | 3R |
| 4F | AAGCATATACGATCCATGCTAACTC | | 4R |
| ***Heading date 3a* gene (*Hd3a*, 4 primer sets)** | |  | |
| 1F | GTACACTGACCGAGCTAAGAGAGAG | | 1R |
| 2F | TGTTTGGTACTTAACCCAAGATGAC | | 2R |
| 3F | AGTGATTTGGAGAAGGATACGC | | 3R |
| 4F | CGTACATGCCAAGTTTGACC | | 4R |
| ***Heading date 6* gene (*Hd6*, 11 primer sets)** | |  | |
| 1F | TTAACAAGGCTCATGCTGTCATAC | | 1R |
| 2F | TACAACTGGCCAGATACTATGTGAA | | 2R |
| 3F | TCTACAGCCTACAAATGTACAAGCA | | 3R |
| 4F | ATCTGCCTTAGTCCGATATCAATAG | | 4R |
| 5F | ATATCCAGACATTGACCTAGACAGG | | 5R |
| 6F | ACTAGGCAATTCAGACTTCAACAGT | | 6R |
| 7F | CAAATGATGCTACTGTTCTTGGCTA | | 7R |
| 8F | CATCATTCTACAGATCCACAGAACA | | 8R |
| 9F | CCAACTTTCCTGACAACTTCATAGT | | 9R |
| 10F | AGAAGATGGGTTGTTAAGGACAGTA | | 10F |
| 11F | CCTACAGCTTGCCATTCACA | | 11F |
| ***Photoperiodic sensitivity 5 (SE5* gene, 9 primer sets)** | |  | |
| 1F | ACTGATAAGCCTGTTTGGAGAATAC | | 1R |
| 2F | CAAATAGAGCTCCTGGACTTACAGA | | 2R |
| 3F | TGCTTCGATAGCTAATATTCTACGC | | 3R |
| 4F | CATTGTTCCATACTTCCATACAGC | | 4R |
| 5F | ACTTAAGTGGAACTGGTCAACTAGC | | 5R |
| 6F | GCATACCAGACCATAGCAGAATAAC | | 6R |
| 7F | ATATTCTGTGCCTGCCAAATG | | 7R |
| 8F | CCAACTTTCATGGGTGTCG | | 8R |
| 9F | ACCACCATCCTCCTGCTACTG | | 9R |
| ***Ealy headingdate 1* (*Ehd1* gene, 11 primer sets)** | |  | |
| 1F | GCTGCTATGTGATATAAGAGGTATCG | | 1R |
| 2F | GTCCATATTCCTGTTTGTCTGAATC | | 2R |
| 3F | CAGTGGATGCACTCTAACTTGAATA | | 3R |
| 4F | GGGAGTACATTAGAGTGATGCAAAT | | 4R |
| 5F | AATTTGATCACTCACTGTCTTCTCC | | 5R |
| 6F | AACTTATTAGGGTGTACTCCGATCC | | 6R |
| 7F | GGTCTCTCTTCTTTCTTCTCCTCAC | | 7R |
| 8F | CATTTCCTCGTCTTCTCCAGTC | | 8R |
| 9F | CTAGTAAGAATGTGTAAGGAG | | 9R |
| 10F | TGTGTGTGTGTGTGTGTAACTTCAT | | 10R |
| 11F | GACTATATTCCATGTGCCTGTTAGC | | 11R |
| ***Ealy headingdate 2* (*Ehd2* gene, 13 primer sets)** | |  | |
| 1F | GATTCACCATGAGGAATACTTTCAC | | 1R |
| 2F | AACTATGTGCTAGCTGATCAATTCC | | 2R |
| 3F | TCTTCATGGCTAGCTTAGAAGTTGT | | 3R |
| 4F | AACAGGAGCTGATTAGGGTTAGG | | 4R |
| 5F | CTTGCCGTATGATTAGCTTAGTTTC | | 5R |
| 6F | GGCTTTGAAATTCTGATGGAGA | | 6R |
| 7F | AACAGTTGGTAGTTGATCAGTGTCC | | 7R |
| 8F | CACACGTACACCCTCTTCCTC | | 8R |
| 9F | CTGATCAGACGAGAGATCAGACAG | | 9R |
| 10F | GTGCATACTTGTTACTCCATCCATC | | 10F |
| 11F | GATCATCACTTAGCTGGATTGGATA | | 11F |
| 12F | TCAACATATGTGTTCTGTAGGCACT | | 12R |
| 13F | CTTTCCGTTAACTTGCAGGTAGTAG | | 13R |
